# Impact of Zr top electrode on tantalum oxide-based electrochemical metallization resistive switching memory: towards synaptic functionalities†

**DOI:** 10.1039/d2ra02456j

**Published:** 2022-05-11

**Authors:** Niloufar Raeis-Hosseini, Shaochuan Chen, Christos Papavassiliou, Ilia Valov

**Affiliations:** Department of Electronics and Electrical Engineering, Imperial College London London SW7 2BT UK n.raeishosseini@imperial.ac.uk; Peter Gruenberg Institute, Research Centre Juelich Juelich 52425 Germany; Institute for Materials in Electrical Engineering II, RWTH Aachen University Sommerfeldstrasse 24 Aachen 52074 Germany

## Abstract

Electrochemical metallization memory (ECM) devices have been made by sub-stoichiometric deposition of a tantalum oxide switching film (Ta_2_O_5−*x*_) using sputtering. We investigated the influence of zirconium as the active top electrode material in the lithographically fabricated ECM devices. A simple capacitor like (Pt/Zr/Ta_2_O_5−*x*_/Pt) structure represented the resistive switching memory. A cyclic voltammetry measurement demonstrated the electrochemical process of the memory device. The *I*–*V* characteristics of ECMs show stable bipolar resistive switching properties with reliable endurance and retention. The resistive switching mechanism results from the formation and rupture of a conductive filament characteristic of ECM. Our results suggest that Zr can be considered a potential active electrode in the ECMs for the next generation of nonvolatile nanoelectronics. We successfully showed that the ECM device can work under AC pulses to emulate the essential characteristics of an artificial synapse by further improvements.

## Abstract 2

Zr is a potential active electrode in the electrochemical metallization cells (ECMs) for the next generation of nonvolatile nanoelectronics. The ECM device works under AC pulses to emulate the essential characteristics of an artificial synapse.

## Graphical abstract

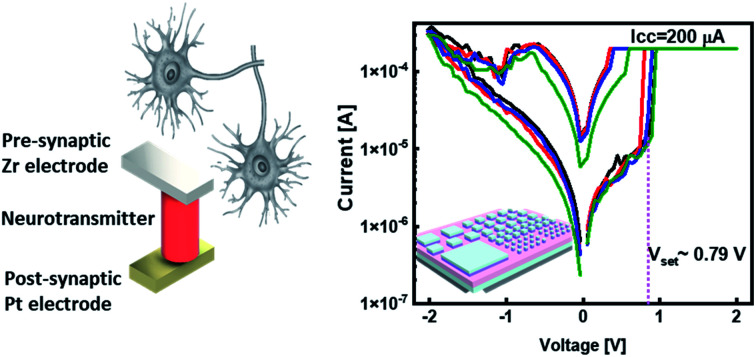

## 1. Introduction

Redox-based resistive switching random access memory (ReRAM) has emerged as a new paradigm for the next generation of nanoelectronic devices; it can bridge the gap between the high speed volatile dynamic random access memories (DRAM) and low-speed nonvolatile flash memory devices.^1–5^ ReRAM operation is based on the resistive switching phenomenon and is a viable candidate for storage class applications, including neuromorphic computing^6^ and logic circuits.^4,7^ It has exceptional merits: fast speed,^8^ high density,^9^ low power consumption,^10^ high performance,^11^ and compatibility with complementary metal-oxide-semiconductor (CMOS) technology.^12–14^ ReRAM has a simple capacitor structure of metal/insulator/metal (MIM), in which the insulator layer is a solid electrolyte (SE).^7^ Various materials, including oxides,^15^ perovskites,^16^ chalcogenides,^7,17^ and polymers^5,12,18,19^ have been used as SE in MIM junctions.

Among the reported SE layer for ReRAMs, transition metal oxides are the most reliable data-storage materials for memory devices due to their high speed,^20^ multilevel switching capability,^21^ and high endurance properties.^22^ HfO_*x*_ and TaO_*x*_ are the most common CMOS-compatible materials. The studies show that each of them can demonstrate tremendous improvement in endurance or retention characteristics of the fabricated memory device, and there is still a trade-off between using these two oxides as a SE in ReRAM structure.^23,24^

Most TaO_*x*_-based RaRAMs work according to oxygen vacancy (V_O_) diffusion inside the solid electrolyte.^25^ In the SE layer of ReRAM, information is saved in a resistance value and applied for external that bias programs the device.^1^ Depending on the material of choice and RS process, ReRAMs are categorized into two distinct types of electrochemical metallization cells (ECMs) and valence change memories (VCMs). ECMs or programmable metallization cells (PMCs) are recognized as conductive bridge random access memories (CBRAMs) or atomic switches.^17^ ECMs operate with electrochemical reactions of active electrodes inside the SE thin film by external electrical stimuli. There is a clear relation between electrochemical properties and the switching performance of ECMs. A conductive filament (CF) is formed and ruptured inside a sandwiched SE between an active and an inert electrode. CF mainly contains atoms of the partially oxidized active electrode under positive voltage.

The TE redox reaction is essential for reliable and reproducible ECM memory.^26^ In ECMs, the choice of the top electrode (TE) material is the most critical parameter for the device operation and performance; it should be an electrochemically active metal. There are many successful studies of ECMs using conventional metal oxides with Ag,^27^ Cu,^28^ Ti,^29^ and alloys of ZrTe/TaN^30^ as active TEs. The electrochemical redox reactions of several active metals have been investigated with cyclic voltammogram and their *I*–*V* characteristics. It is concluded that compared to the conventional TEs of ECM, Zr is a less favorable choice of material because of its high affinity to oxygen and producing a robust passivating oxide based on Gibbs free energy of formation for metal/metal-cation combinations.^1^

In this work, we aim to go through the impact of the Zr electrode deeply and empirically investigate the potential possibility for utilization of the Zr active electrode in a Ta_2_O_5_-based ECM device. Zirconium (Zr) is a solid CMOS-compatible transitional metallic element containing hafnium and excellent corrosion resistance properties. Zr is produced by an electrochemical reduction of zirconia and hafnia. Due to its exceptional corrosion resistance against alkalis, acids, and other agents, its maintenance is more cost-effective than other metals. In addition, it has the merits of non-toxicity and biocompatibility, which makes it a suitable element in chemical processing and biocompatible devices.^31^ Therefore, we are interested in studying the resistive switching properties of ReRAM using Zr for future replacement of conventional metals such as Al, Cu, and Ag in ECM memory cells. It has been proved that introducing Zr in TaO_*x*_-based ReRAM is favorable for developing the essential characteristics of the memory device. Zr doping leads to improving retention and endurance and reducing forming voltage. The Zr-doped devices illustrate enhanced stability of the conductive filament (CF).^32^

Inspired by the idea of ion implementation of Zr in ReRAM structure to get a high-performance memory device and achieve a robust endurance using ZrN_*x*_ as the electrode material of HfO_*x*_-based ReRAM,^33^ we study the effect of Zr as a TE and its possible replacement by conventional metals. Since most material selections for ECMs are based on empirical observations, we examine the electrochemical characteristics of Zr in a Pt/Zr/Ta_2_O_5−*x*_/Pt system. We investigate the electrochemical reaction of the ECM system using a cyclic voltammogram (CV). Moreover, we compared the effect of conventionally used active and inert electrodes on the TaO_*x*_-based ReRAMs in both ECM and VCM memories (ESI, Tables 1 and 2†). We confirm that Zr as a TE attends the redox process and diffuses into the SE by applying voltage with fast passivation and subdued current density. Based on the experimental data, Zr can be a potential alternative to serve as a TE in ECMs. Considering our device an artificial synapse, we emulate essential functions of biological synapse such as excitatory postsynaptic current (EPSC) and paired-pulse facilitation (PPF). Moreover, a successful linear synaptic behavior is achieved by successive neural spikes. The demonstrated results in the ECM are suitable for future applications in neuromorphic computing.

## 2. Results and discussion

### 2.1. Device structure

**Fig. 1:**
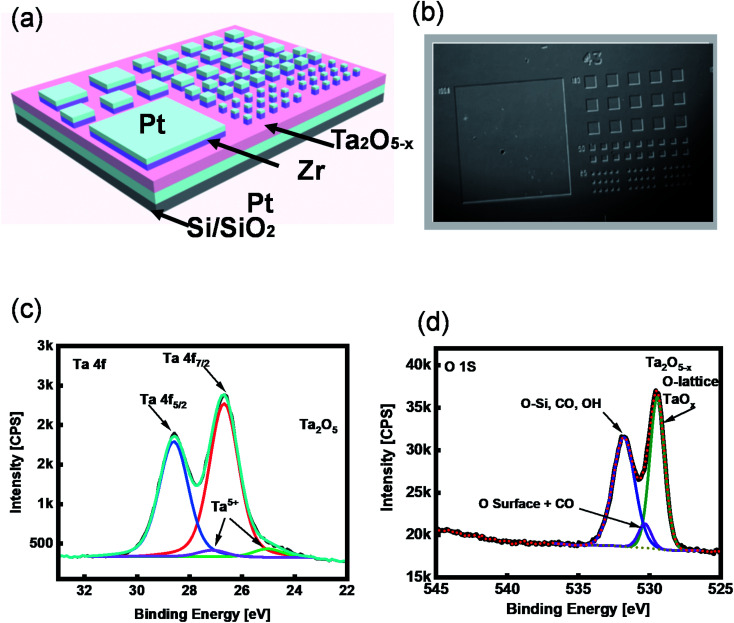
(a) Schematic illustration of the ECM memory device with Ta_2_O_5−*x*_ solid electrolyte (SE) and Zr/Ta_2_O_5−*x*_/Pt/SiO_2_/Si structure; the ECMs are with various dimensions (2 μm to 500 μm). (b) Optical image of the devices fabricated by photolithography. (c) XPS core level of Ta 4f and (d) O 1s.

The memory effect of the tantalum oxide-based ReRAM was studied using 10 nm Ta_2_O_5−*x*_ thin-film as an insulator layer. The whole device stack was fabricated on a p-doped SiO_2_/Si substrate. The sub-stoichiometric thin film was deposited by reactive radiofrequency sputtering with an optimized Ar and O_2_. Before growing the active layer of ReRAM, 50 nm Pt was sputtered on the substrate to serve as a bottom electrode. An optical lithography and liftoff process were performed to pattern the top electrodes. The fabrication was accomplished by sputtering 15 nm Zr with Pt capping layers. A Pt/Zr/Ta_2_O_5−*x*_/Pt structure was used to demonstrate the ReRAM with a two-terminal metal–insulator–metal structure (Fig. 1a and b).

### 2.2. XPS characterization

To investigate the chemical composition of the tantalum oxide-based SE, we characterized the as-deposited Ta_2_O_5−*x*_ thin film using X-ray photoelectron spectroscopy (XPS), where the peaks of Ta 4f and O 1s are demonstrated (Fig. 1c and d). The peak ratio of Ta binding energy (B.E.) is 2 : 5, Ta 4f_7/2_ and Ta 4f_5/2_ exist at the peak values of 25.39 eV and 27.29 eV, respectively. These peak values are related to Ta_2_O_5_ (Fig. 1c). The XPS BE peak of oxygen at ∼529 eV represents the existence of Ta_2_O_5−*x*_ in a lattice of tantalum oxide (Fig. 1d).

### 2.3. Electrochemical characterizations of the memory device

#### 2.3.1. Cyclic voltammetry

**Fig. 2:**
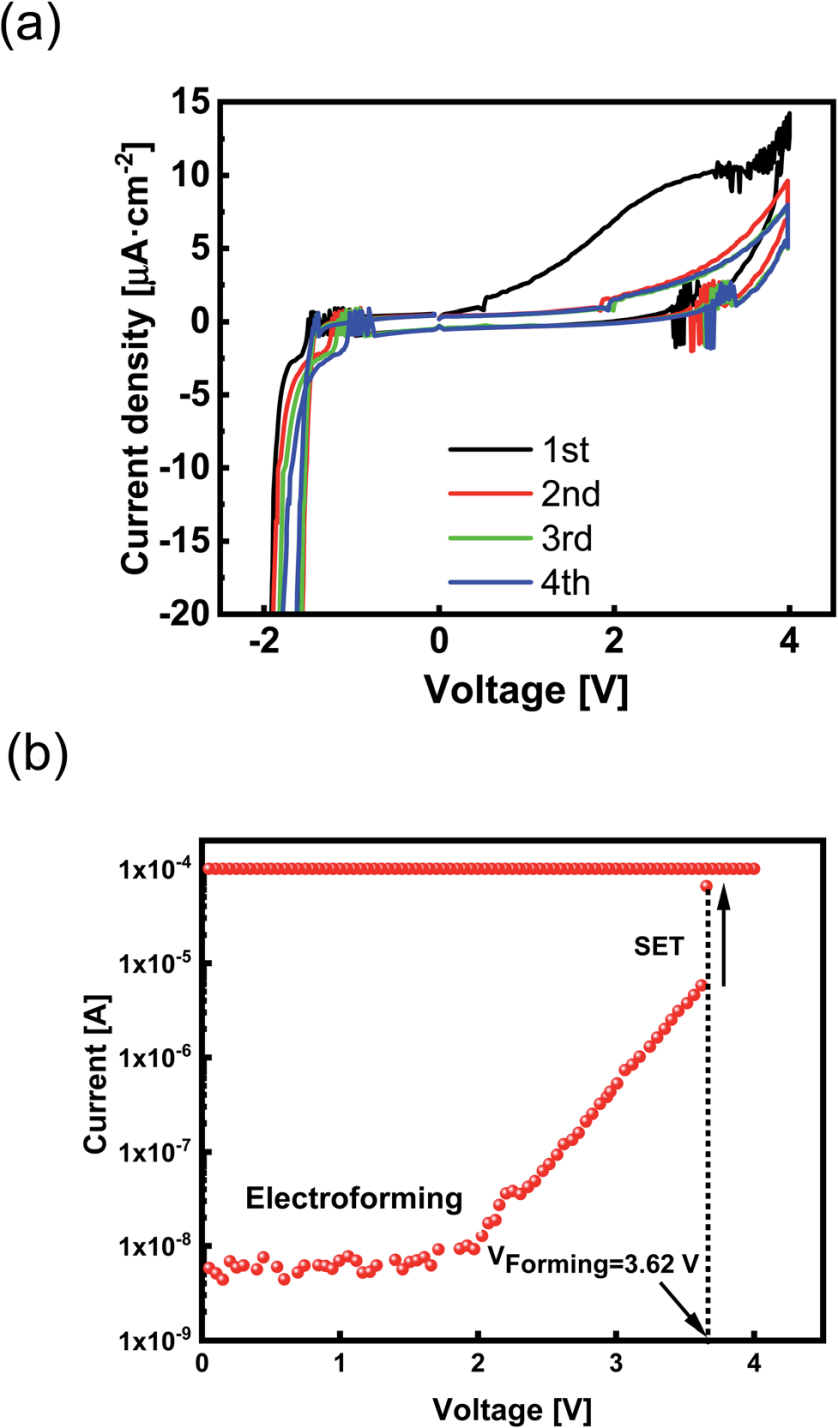
(a) Cyclic voltammogram of Zr/Ta_2_O_5−*x*_/Pt/SiO_2_/Si memory device. (b) The typical forming curve of Zr/Ta_2_O_5−*x*_/Pt/SiO_2_/Si device with a sudden current transition where the device turns in the ON state. Current compliance of 100 μA was imposed to avoid the complete breakdown of the device.

In ECM cells, the switching phenomenon depends on redox processes and CV, which is an essential characteristic of their oxidation/reduction (redox) process. Therefore, we studied the redox reactions in the Ta_2_O_5_-based ECM device using CV. We believe that ECM is the dominant mechanism, and so we exclude oxygen vacancy-driven CFs in the CV measurement. To perform CV measurement, we applied a positive voltage sweep to the TE (Pt coated Zr) before reaching the electroforming process. During CV measurement, Ta_2_O_5−*x*_ acts as a solid electrolyte, and Pt is the counter electrode; the current response traces the redox reactions of the active Zr electrode to the sweeping voltage. The CV was recorded by applying 4 V positive bias to the TE and oxidation of Zr and sweeping by negative bias to −2 V and reduction of TE. Through voltage sweeping, the positive/negative current peak corresponds to the redox process of the Zr top electrode. A standard current density peak in cyclic voltammogram is consistent with redox processes at the interfaces.^1^ The oxidation peak in positive bias indicates the formation of Zr^4+^. The redox behavior is reversible and current peaks are decreased by increasing the number of cycles (Fig. 2a).

Each peak's corresponding current and voltage indicate the redox reaction rate and the required thermodynamic force.^1^ Two typical current density peaks are discovered at −1.5 V and + 2.7 V in the CV diagram (Fig. 2a). A broad oxidation peak at *V*_Oxidation_ ≈ + 2.7 V indicates the formation of Zr^4+^ according to Zr ↔ Zr^4+^ + 4e^−^ reaction. A significant reduction peak at *V*_Reduction_ ≈ −1.5 V has appeared by reversing the applied potential. Because Zr is a transition metal with high oxygen affinity, the formation of highly passivating oxide is inevitable.

Although the oxidized Zr electrode makes an interfacial barrier layer, it is thin enough to permit ion movement. By further cycles of voltage sweeping, the current density decreases (Fig. 2a). The lower current density at second and third sweeps is due to the passivation process that affects the current response at the CV diagram. The passive layer of Zr oxide works as an extra series resistor in the electrochemical circuit^1^ and prevents diffusion of Zr^4+^ into the SE layer.

#### 2.3.2. Electroforming process

An electrical bias was applied on the top electrode (TE) to switch the highly insulating fabricated device from its pristine state to a set process. This electroforming step is required to change the device from a high resistance state (HRS) to a low resistance state (LRS) for the first time. The first voltage sweep that leads the devices to be formed is forming voltage (*V*_Forming_) (Fig. 2b). We used a current compliance limit (*I*_CC_) of 200 μA to prevent the permanent breakdown of the ReRAM devices during the positive voltage sweeps. After forming voltage, the devices can work adequately toggling between negative and positive biases. During the positive voltage sweep, the current abruptly increased to 100 μA (*I*_CC_) at a specific voltage of *V*_Forming_ (3.62 V); therefore, the devices are formed to get switched to be ON at their LRS state (Fig. 2b).

### 2.4. Electrical characterizations

**Fig. 3:**
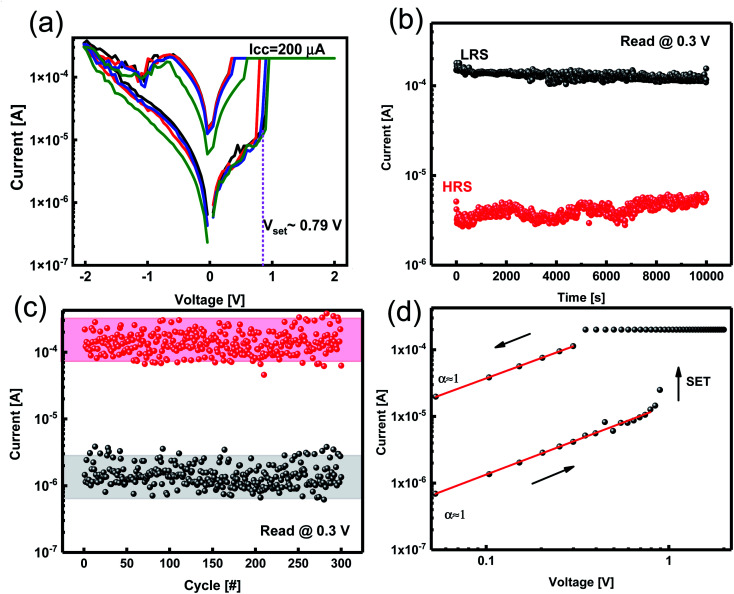
ReRAM characteristics of the fabricated devices. (a) Log-scale *I*–*V* characteristics for the Zr/Ta_2_O_5−*x*_/Pt/SiO_2_/Si ReRAM showing reproducible resistive switching behavior. The compliance current of 200 μA is induced to prevent the permanent breakdown of the device. (b) Retention test by measured LRS and HRS resistance values (read at 0.3 V); (c) endurance properties of the device by applying 10 μs set and reset pulses with 0.3 V reading voltage. (d) Double logarithmic *I*–*V* curve of positive bias showing linear ohmic conduction mechanism.

We measured the current–voltage (*I*–*V*) characteristics of ReRAM devices under ambient temperature. The Ta_2_O_5−*x*_-based ReRAMs showed the typical bipolar resistive switching (RS) property of ECM types (Fig. 3a). The voltage as a thermodynamic-driving force was applied to the Zr active electrode while the Pt bottom electrode was grounded. Considering an electroforming step initiated at +4 V, the devices were activated and, therefore, switched between the LRS and the HRS (Fig. 3a).

*I*–*V* responses of the Pt/Zr/Ta_2_O_5−*x*_/Pt devices were measured under dc sweeping voltage applied as 0 V → 2 V → 0 V → −2 V → 0 V to the TE (Zr). In the first positive voltage sweep from 0 to *V*_set_ ∼ 0.79 V, Zr (TE) oxidized and changed to Zr^4+^; the cations formed a conductive filament (CF) at the interface of the Pt (BE) by reducing to Zr atoms. After the formation of CF, the insulating solid electrolyte was changed to be the LRS. We applied a compliance current of 200 μA to avoid device breakdown. By changing the polarity of the applied voltage, the CF was ruptured, and the ReRAM returned to the HRS. We measured the electrical characteristics of the ECM device with different top electrodes of Al, Ag, and Cu (ESI Fig. S1†).

To evaluate the stability of the ReRAM, a data retention test was conducted. The data retention measurement was performed for both the LRS and HRS states of the Zr/Ta_2_O_5−*x*_/Pt device with a reading voltage of 0.3 V. A high enough ON/OFF ratio of ∼10^2^ was obtained, which is comparable with previous studies, and no appreciable degradation in the current of LRS and HRS was observed (Fig. 3b). It has been reported that by doping gadolinium in the Ta_2_O_5_ structure, the retention has been improved and extrapolated time retention for more than 10 years has been achieved.^34^

We assessed the reliability of the Ta_2_O_5−*x*_-based ReRAM by endurance characteristics for 100 cycles under 0.3 V reading bias. This stability indicates the robust memory property of the ReRAM devices with Zr electrodes (Fig. 3c). To explain the conduction mechanism, we redrew double logarithmic plots of the *I*–*V* curves for positive voltage sweeps (Fig. 3d). The switching mechanism is assigned to forming and breaking a conductive filament (CF).

We applied positive bias to ECMs, and the *I*–*V* curve was linear until reaching “set” voltage, which is a characteristic of ohmic conduction. The linear relation between current and voltage indicates that Zr CFs construct in both LRS and HRS states (Fig. 3d). Therefore, the redox reaction of the Zr filaments is supported by the conduction mechanism of filamentary switches.

To realize a high-performance Schottky barrier diode, a high-quality Schottky contact with large Schottky barrier heights (*Φ*^n^_B_) and low leakage current is essential.^35^

The difference in the barrier height between the ReRAM switching oxide (Ta_2_O_5_) and the metal has been achieved using the core-level alignment method. The hole barrier height, *Φ*^p^_B_, and Schottky barrier height, *Φ*^n^_B_ at the Zr/Ta_2_O_5_ and Pt/Ta_2_O_5_ interface are shown by a correlation between valence band maximum and core-level energies of Ta_2_O_5_ (ESI S2†). Where, *Φ*_B_ is the Schottky barrier height, and *χ* is the electron affinity of Ta_2_O_5_.^36^

### 2.5. Resistive switching mechanism

The switching mechanism of ReRAM highly depends on the top electrode material. However, in ReRAMs with Ta_2_O_5−*x*_/TaO_*x*_ active layer cooperation between metallic and hopping conduction is a possible RS mechanism.^37^

Considering the electrical properties, the device operation is due to redox reactions of field-induced Zr ions, which move through the SE layer. The first-principles calculations have proved that the effect of interstitial Ta (Ta_i_) is vital for the RS mechanism. In addition, it is shown that the presence of an electric field in ReRAMs leads to the movement of Ta_i_ because of its higher charge states compared with V_O_. Based on the calculations, Ta cations play a vital role in TaO_*x*_-based ReRAMs in both VCMs and ECMs.^25^

**Fig. 4:**
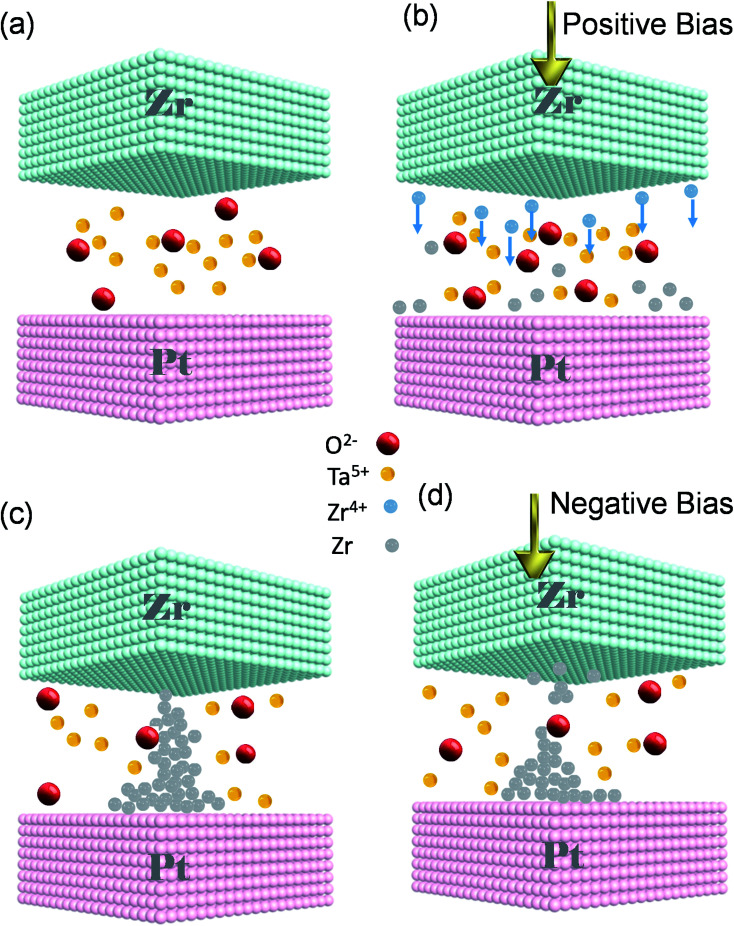
(a) Proposed operation mechanism of the electro metallization cell. (a) Pristine state. (b) Set process; Zr^4+^ ions are reduced to Zr^0^ at the top electrode; ion movement under positive bias voltage; (b) reducing of Zr cations which provide a source for filament formation. (c) The Zr-based conductive filament is formed and causes a connection between two electrodes. (d) OFF state; Zr filament ruptures in the solid electrolyte under negative bias.

We propose that the switching mechanism is related to the creation and rupture of a CF inside SE (Fig. 4a–d). We consider the ReRAM as an electrochemical cell in which Zr is an anode and Pt is a cathode of the cell while the insulator serves as a SE layer. Without any external bias, the device is in its pristine state (Fig. 4a). Zr TE works as the anode of an electrochemical cell. The electric field is elevated by applying a positive voltage to the TE, and Zr is oxidized to Zr^4+^. Anodic dissolution (oxidation) of Zr occurs at the interface of TE and the SE layer (Zr → Zr^4+^ + e^−^). The Zr^4+^ cations move downward through the tantalum oxide thin film (Fig. 4b). Due to the field's accelerated transport, the formed cations migrate through the SE toward the Pt inert electrode.^1,12^ Conversely, a cathodic deposition reaction happens at BE; Pt is responsible for the cathodic reaction of our cell. When the cations arrive at the interface of the BE and SE, they are reduced to Zr atoms (Zr^4+^ + 4e^−^ → Zr). Afterward, the Zr atoms are electrodeposited on the Pt counter electrode and gathered to make Zr CFs upward to the TE. Then, a short circuit is made up by connecting the CF to the TE, and the device changes its state to LRS (Fig. 4c). To switch the ECM to its pristine condition, we reversed the polarity of the applied bias. Applying an adequate negative bias leads to breaking the self-assembled CF (Zr → Zr^4+^ + 4e^−^). The reduction reaction of the mobile Zr^4+^ cations takes place while moving towards TE (Zr^4+^ + 4e^−^ → Zr) and the device returns to its initial HRS state (Fig. 4d). The explained switching characteristic mechanism is depicted step by step in a linear *I*–*V* curve (Fig. S3, ESI†). The performed tests indicate that the Pt/Zr/Ta_2_O_5−*x*_/Pt device is a reliable and reproducible ECM type ReRAM. Changing the device size affects the size and shape of the conductive filament; without affecting the conduction mechanism.^38^

### 2.6. Towards synaptic functionalities

**Fig. 5:**
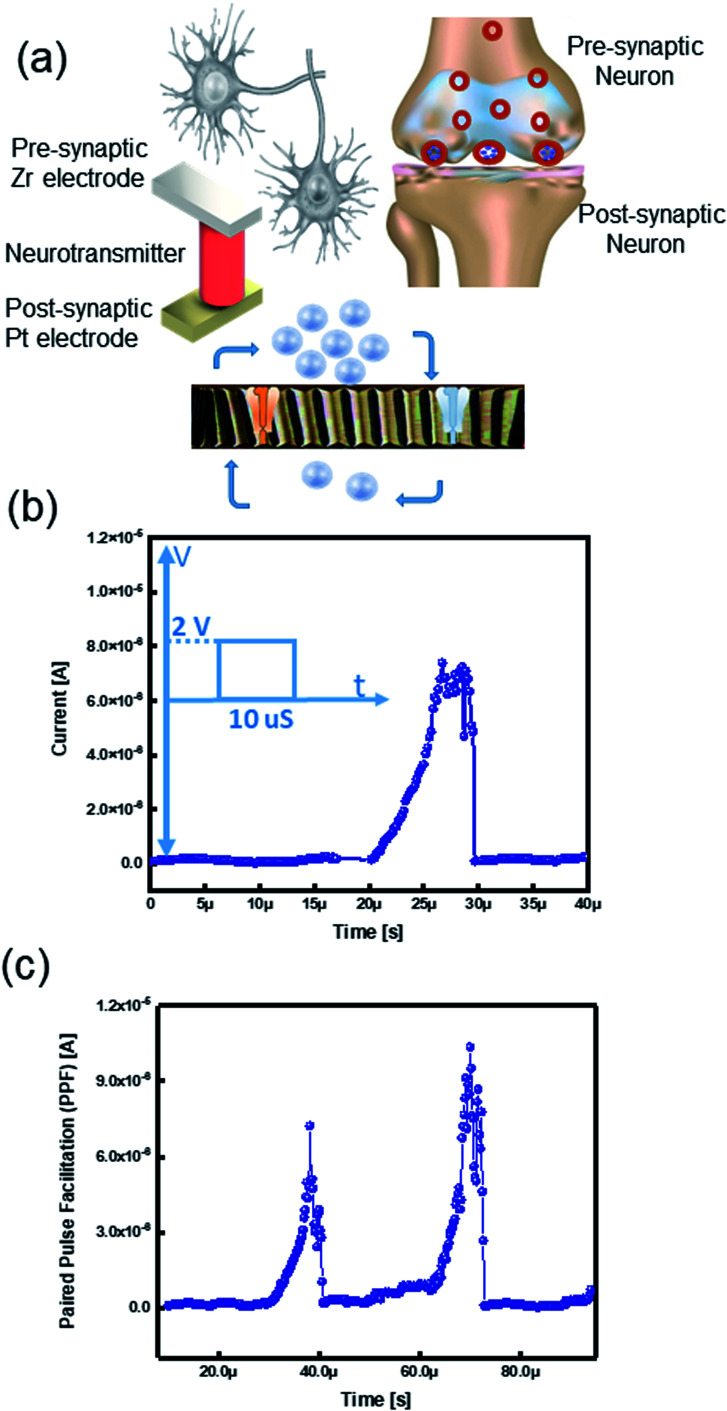
Response of artificial synapse to the applied pulse. (a) Schematic illustration of synapse and its behavior. (b) Excitatory postsynaptic current (EPSC); inset: EPSC triggered by a presynaptic spike (2 V, 10 μs) at reading bias 0.3 V. (c) Synaptic improvement attained by two sequentially applied pulses, imitating a biological process of PPF; the EPSC triggered by two spikes with an interspike interval time of 20 μs.

The human brain processes data by using a massive amount of neurons and their synaptic connections.^39,40^ Comprehension and learning process occurs *via* thousands of synaptic connections between neurons.^41–44^ Pre-synaptic spikes stimulate various synapses; these signals are diffused through neurotransmitters and excite output signals, namely post-synaptic spikes (Fig. 5a).^6^ Herein, we examine the possibility of assimilation of our memory device as a brain-inspired artificial synaptic device to realize a neuromorphic computing system.

#### 2.6.1. Effect of pulse scheme on triggering the devices

We swept positive and negative voltages to form a CF between electrodes. However, applying a train of input pulses is another valuable method to create a CF between the anode and cathode of ECM. Like a biological synapse, which functions based on the transmission of Ca^2+^ cations throughout the ion channel,^45^ our device responds to the applied spikes by Zr^4+^ cations. Therefore, we manipulate the Zr dynamics of CF to emulate the Ca^2+^ dynamics of a biological synapse (Fig. 5a).

##### 2.6.1.1. Excitatory post-synaptic current (EPSC)

Excitatory postsynaptic potential (EPSP) or synaptic strength is defined by *G* conductance. The total conductance is determined by summation of the conductance without a presynaptic spike (*G*_0_). The conductance change results from the pre-synaptic spike (Δ*G*).^6^ EPSP is an essential synaptic function suggested as a temporary current produced by ion flows from pre-synapse to post-synapse.^46^ Considering the TE and BE as the presynaptic and postsynaptic terminals, the insulator layer works as a neurotransmitter (Fig. 5a). To emulate the EPSC characteristics in a biological synapse, a pre-synaptic voltage spike was applied to the TE that causes movement of the ions into the postsynaptic neuron. The voltage spike (2 V, 10 μs) triggered an EPSC; therefore, the current level was momentarily increased while applying the voltage spike and then steadily decreased to the original current level later on by removing the pulse (Fig. 5b). Since the ions migrate within a short distance, they quickly return to their initial state.^2,47^ We postulate the ongoing relaxation process and the remained nonvolatile charge in the memristive device to ionic charges in the SE of the ECM cell.^6,48^

##### 2.6.1.2. Paired pulse facilitation (PPF)

In a biological brain, the learning procedure happens by synaptic plasticity categorized into short-term- (STP) and long-term-plasticity (LTP).^49^ Data processing and calculations are performed by STP, whereas interpreting temporal data is governed by paired pulse facilitation (PPF). In the human brain system, PPF is a form of STP that is a progress of the reaction to a pair of pulses arrived in a fast sequence.^50–52^ The ReRAM device mimicked the (PPF) properties of the biological synapse, which is an increase in response for the second pulse when a pair of spikes arrive with a time interval (<1 s).^53^ Therefore, in the PPF phenomenon, the postsynaptic response is improved when the second spike follows the previous one instantaneously.^41,53,54^ We applied two successive presynaptic spikes (2 V, 10 μs) on Zr active electrode with a 20 μs interval. When the second voltage spike was applied, it triggered the EPSC by a higher current level change than the former spike (Fig. 5c). Because of the correlation between voltage and time, the increasing number of the input spikes in an advancing scheme is similar to triggering the device with a higher voltage.^55^ The elevated current level is ascribed to an inadequate time interval between two pulses, which the device cannot relax to its pristine state.^6^

##### 2.6.1.3. Potentiation and depression with a linear synaptic weight

**Fig. 6:**
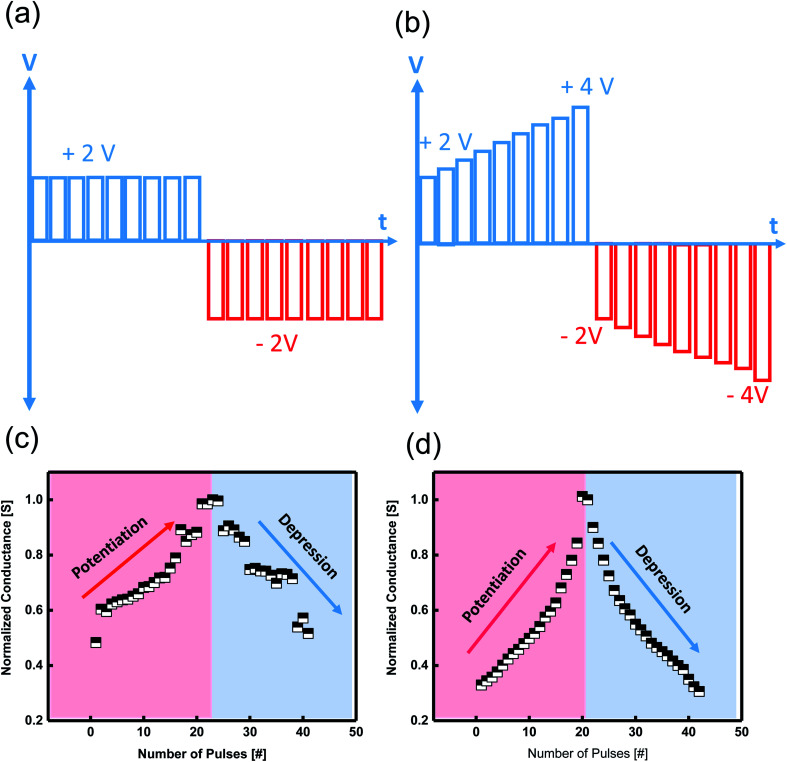
Various programming pulse schemes. (a) Successive identical and (b) non-identical neuron spikes. (c and d) Normalized potentiation and depression properties of TaO_2−*x*_ thin-film memristor with two different pulse schemes. The non-identical spikes lead to a linear synaptic behavior with a slope of 1. Using nonidentical presynaptic spikes, a significant improvement in symmetry was observed.

The current response of the synaptic device to a series of positive and negative pre-spike pulses shows potentiation and depression. Triggering the device by applying vigorous positive/negative AC pulses leads to an increase/decrease of synaptic conductance leading to potentiation and depression of the artificial synapse. The tuning conductance is representative of a biological synapse known as synaptic weight update.^56^ We applied several twenty identical pulses with a pulse width of 10 μs and an amplitude of 2 V to initiate potentiation, followed by another 20 pulses with an amplitude of −2 V to cause depression. We applied a sum of 40 pairs of identical positive/negative pulse trains to get the potentiation/depression (Fig. 6a). The more nearly linear the conductance, the better the device emulates the human brain's abilities. To realize the linear conductance behavior of the device, we applied 20 non-identical pulse strains with a gradual increase from 2 V to 4 V. The same scheme was applied for the negative non-identical pulses (Fig. 6b). By engineering the input spikes, the device demonstrated linear potentiation/depression property, an ideal property of an artificial synapse. A comparison between the conductance response of the device based on pulse schedules is illustrated in Fig. 6a and b.

## 3. Conclusions

We fabricated and characterized electrochemically active resistive switching memory (ReRAM). Zr and Ta_2_O_5−*x*_ are introduced as the active electrode and resistive switching layer of electrochemical metallization memory (ECM), respectively. The ECM type non-volatile memories has a simple (Pt/Zr/Ta_2_O_5−*x*_/Pt) structure. The devices operate with a forming voltage and reliable bipolar resistive switching properties. Furthermore, the fabricated ECMs showed a significant memory performance with a good ON/OFF ratio (∼10^2^) and reasonable data retention time (∼10^4^ s). Based on the empirical data, we conclude that the electrochemical reactions inside the oxide film are responsible for the formation and rupture of a Zr-based conductive filament. This study demonstrates the possibility of using Zr as a top electrode of oxide-based ECMs type ReRAMs. We successfully showed that the ECM device can work under an AC pulse to emulate the essential characteristics of an artificial synapse by further improvements. Moreover, the engineered pre-and post-spikes using non-identical pulses potentiated and depressed the synaptic device in a linear conductance fashion.

## 4. Methods

### 4.1. Device fabrication

The tantalum oxide-based ECM devices with a stacked structure of Pt/Zr/Ta_2_O_5−*x*_/Pt were fabricated on a thermally oxidized p-doped (100)-oriented Si wafer with 430 nm of SiO_2_. We sputtered 50 nm Pt bottom electrode with 10 nm TiO_*x*_ (adhesion layer) on top of the Si/SiO_2_ substrate. One-third of the Pt layer was covered with a standard photoresist for a further lift-off. Before thin film deposition, we put the base pressure of the sputtering chamber lower than 10^−5^ mbar. We deposited the metallic layers at room temperature in an Ar atmosphere. 10 nm of Ta_2_O_5−*x*_ sub-stoichiometric thin film was deposited from a Ta target using radiofrequency (RF) magnetron sputtering in a reactive atmosphere composed of a mixture of Ar and O_2_ by a tuned operating pressure and RF power.

We used conventional UV photolithography using a customized optical mask following a lift-off process to pattern the top electrodes of ReRAM devices with different sizes ranging from 25 × 25 μm^2^ up to 1 × 1 mm^2^. The fabrication was fulfilled by deposition of 15 nm Zr as the top electrode. We used 30 nm Pt as a capping layer to avoid oxidation of the bottom electrodes.

### 4.2. Electrical and electrochemical characterization

Electrical measurements were executed in two-terminal configurations using a semiconductor device parameter analyzer (Keysight, B1500A) at atmospheric pressure and ambient temperature. We used 100 × 100 μm^2^ devices for the measurement. The Zr electrodes were biased in all the measurements while the Pt electrode was grounded. Cyclic voltammetry measurements were performed by Keithley 6430 sub-femtoampere source meter.

## Conflicts of interest

There are no conflicts to declare.

## Supplementary Material

RA-012-D2RA02456J-s001
