# Supplementary material for: Impact of Zr top electrode on tantalum oxide-based electrochemical metallization resistive switching memory: towards synaptic functionalities
Source: RSC Adv. 2022 May 11;12(22):14235–45. doi: 10.1039/d2ra02456j (PMC9092617; doi:10.1039/d2ra02456j)
Supplement: RA-012-D2RA02456J-s001 [file RA-012-D2RA02456J-s001.pdf]

Electronic Supplementary Material (ESI) for RSC Advances.
This journal is © The Royal Society of Chemistry 2022

Supporting Information

Impact of Zr Top Electrode on Tantalum Oxide-based Electrochemical Metallization Resistive Switching Memory (ReRAM): Towards Synaptic Functionalities

Niloufar Raeis-Hosseini[1], Chen Shauchuan[2], Christos Papavassiliou[1], and Ilia Valov[2,3]*

1 Department of Electronics and Electrical Engineering, Imperial College London, London, SW7 2BT, United Kingdom.

2 Peter Gruenberg Institute, Research Centre Juelich, Juelich 52425, Germany.

3 Institute for Materials in Electrical Engineering II, RWTH Aachen University, Sommerfeldstrasse 24, Aachen 52074, Germany.

* Corresponding author: n.raeishosseini@imperial.ac.uk

The structure of the memory device	Tantalum Oxide form	ECM-based ReRAM	Top Electrode Material	XPS data available	Retention & Endurance	Cyclic Voltammetry Measurement	Bipolar Polarity	Voltage (V)	Current Level (mA)	On/Off Ratio	Reference
$Pt/Ta/Ta_2O_5/Pt$	TaO_x (Ta_2O_5)	No	Ta	No	No	No	Yes	-1.5, 1	1.5	No	[1]
$Ta/(TaO_2)Ta_2O_5/Pt$	TaO_x (Ta_2O_5)	No	Ta	No	No	No	Yes	-3, 2	60	No	[2]
$Pd/Ta_2O_{5-x}/TaO_y/Pd$	Ta_2O_{5-x}/ TaO_y	No	Pd	No	Yes	No	Yes	-2, 2	2	Yes	[3]
$Pt/ Ta_2O_{5-x}/Ta$	Ta_2O_{5-x}	No	Pt	Yes	Yes	No	Yes	-1, 2	1	Yes	[4]
$Ti/Ta_2O_{3-x}/Pt$	Ta_2O_{3-x}	No	Ti	Yes	Yes	No	Yes	-1.5, 1.5	3	No	[5]
$Ta/Ta_2O_5/Pt$	Ta_2O_5	No	Ta	No	No	No	×	×	×	No	[6]
$Pt / Zr /Ta_2O_{5-x} / Pt$	Ta_2O_{5-x}	Yes	Zr	Yes	Yes	Yes	Yes	-2, 2	0.2	Yes	This work

Supporting Table 1. Comparison of different inert top electrode materials on the electrical properties of TaO_x-based VCM ReRAMs.

The structure of the memory device	Tantalum Oxide form	ECM-based ReRAM	Top Electrode Material	XPS data available	Retention & Endurance	Cyclic Voltammetry Measurement	Bipolar Polarity	Voltage (V)	Forming Voltage (V)	Current Level (mA)	On/Off Ratio	Reference
$Cu/Ta_2O_5/Pt$	TaO_x (Ta_2O_5)	Yes	Cu	No	No	No	Yes	-3, 3	6	100	No	[7]
$Cu/TaO_x/Pt$	TaO_x	Yes	Cu	No	No	No	No	No	No	compare	No	[8]
$Ag/ TaO_x/TiN$	TaO_x	Yes	Ag	No	No	No	Yes	-1, 1	No	1	No	[9]
$Ag/TaO_x/Pt$	TaO_x	Yes	Ag	No	No	No	Yes	-.1, .15	No	3	No	[10]
$Al/TaO_x/Pt$	TaO_x	Yes	Al	Yes	No	No	Yes	-15, 15	Yes	1.2n	No	[11]
$Pt / Zr /Ta_2O_{5-x} / Pt$	Ta_2O_{5-x}	Yes	Zr	Yes	Yes	Yes	Yes	-2, 2		0.2	Yes	This work

Supporting Table 2. Comparison of different active top electrode materials on the electrical properties of TaO_x-based ECM ReRAMs.

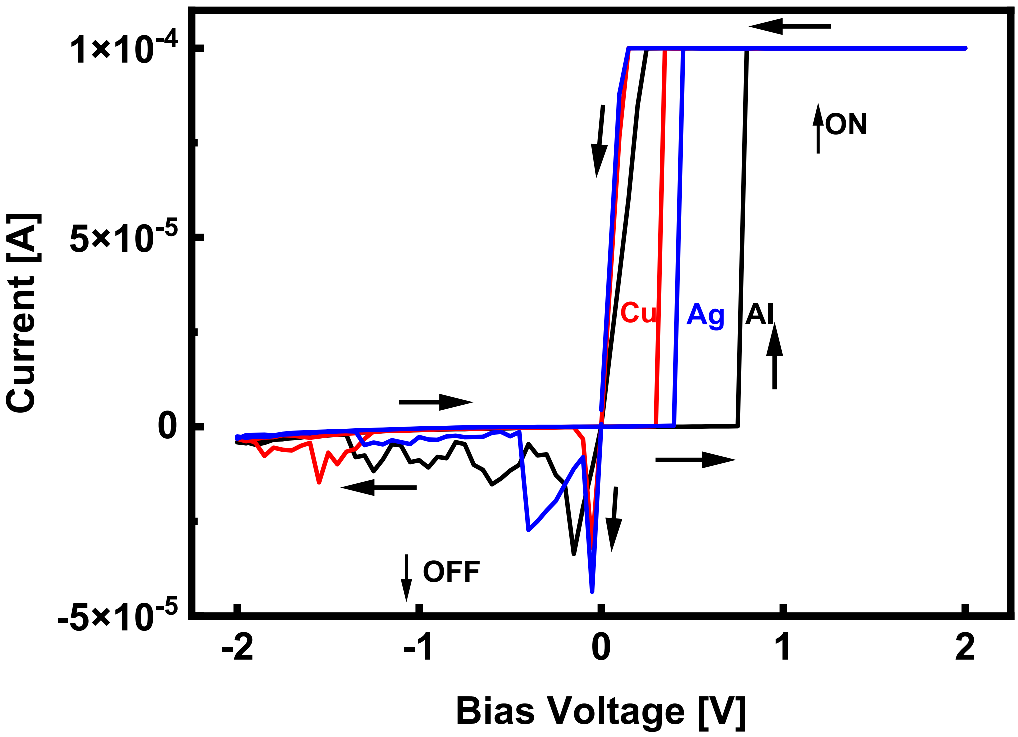

Supporting Figure S1| *I–V* characteristics of the resistive switching memory device with different top electrodes of Al, Ag, and Cu.

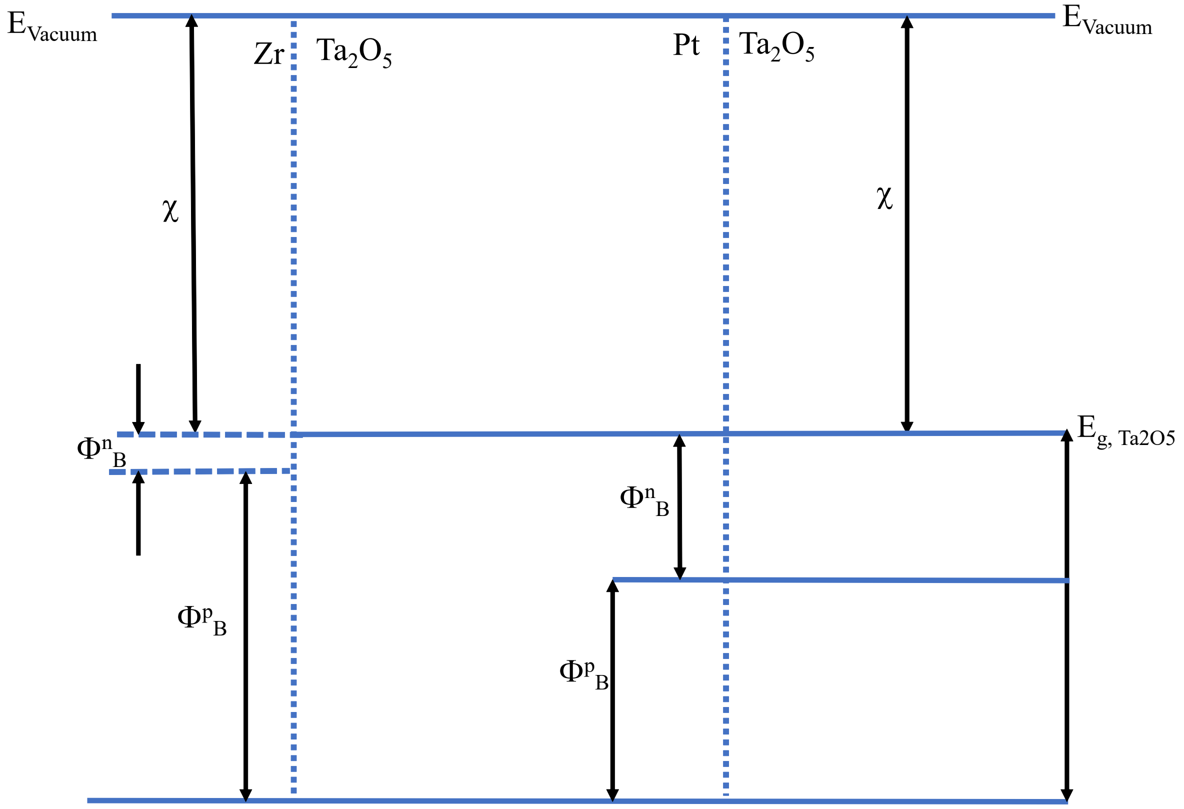

Supporting Figure S2| Energy band diagram for Zr and Pt electrodes in contact with Ta_2O_5 insulator layer.

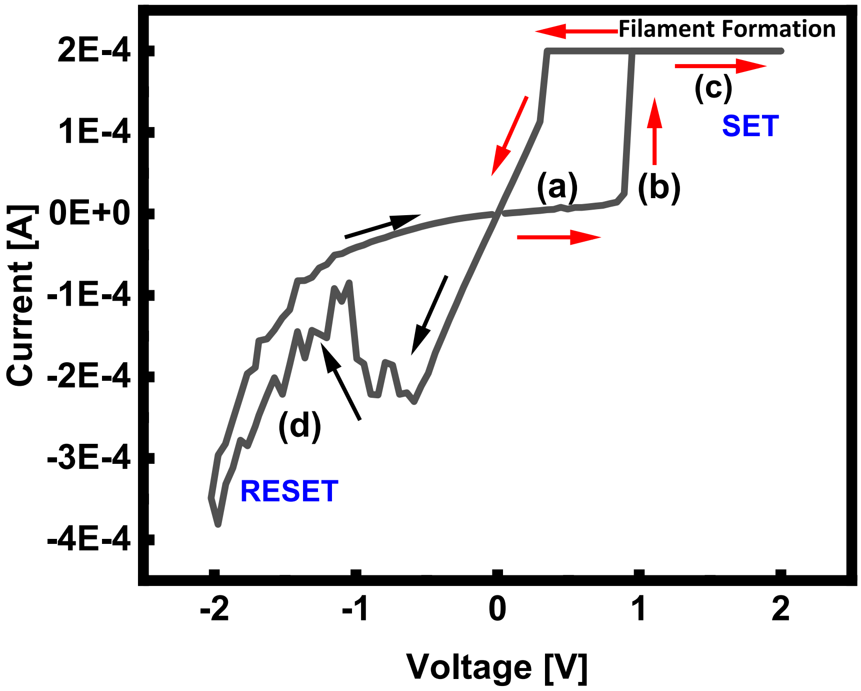

Supporting Figure S3| linear *I–V* characteristics of the resistive switching memory device

References:

1 Rosário, C. M. *et al.* Correlation between the transport mechanisms in conductive filaments inside Ta2O5-based resistive switching devices and substoichiometric TaOx thin films. *Applied physics letters* **112**, 213504 (2018).

2 Wedig, A. *et al.* Nanoscale cation motion in TaO x, HfO x and TiO x memristive systems. *Nature nanotechnology* **11**, 67-74 (2016).

3 Yang, Y., Sheridan, P. & Lu, W. Complementary resistive switching in tantalum oxide-based resistive memory devices. *Applied Physics Letters* **100**, 203112 (2012).

4 Skaja, K. *et al.* Reduction of the forming voltage through tailored oxygen non-stoichiometry in tantalum oxide ReRAM devices. *Scientific reports* **8**, 1-7 (2018).

5 Abbas, Y. *et al.* Compliance-free, digital SET and analog RESET synaptic characteristics of sub-tantalum oxide based neuromorphic device. *Scientific reports* **8**, 1-10 (2018).

6 Zhu, L., Zhou, J., Guo, Z. & Sun, Z. Synergistic resistive switching mechanism of oxygen vacancies and metal interstitials in Ta2O5. *The Journal of Physical Chemistry C* **120**, 2456-2463 (2016).

7 Tsuruoka, T., Terabe, K., Hasegawa, T. & Aono, M. Forming and switching mechanisms of a cation-migration-based oxide resistive memory. *Nanotechnology* **21**, 425205 (2010).

8 Li, Y., Tsurumaki-Fukuchi, A., Arita, M., Morie, T. & Takahashi, Y. in *2019 Silicon Nanoelectronics Workshop (SNW).* 1-2 (IEEE).

9 Pyo, J. & Kim, S. Non-volatile and volatile switching behaviors determined by first reset in Ag/TaOx/TiN device for neuromorphic system. *Journal of Alloys and Compounds* **896**, 163075 (2022).

10 Huang, X. *et al.* Configurable ultra-low operating voltage resistive switching between bipolar and threshold behaviors for Ag/TaOx/Pt structures. *Applied Physics Letters* **113**, 112103 (2018).

11 Liu, Q., Xue, Q., Wang, Y., Wei, X. & Hao, J. Bifunctional Device with High-Energy Storage Density and Ultralow Current Analog Resistive Switching. *Advanced Electronic Materials* **7**, 2000902 (2021).
